# Fabrication, characterization, and biological evaluation of anti-HER2 indocyanine green-doxorubicin-encapsulated PEG-b-PLGA copolymeric nanoparticles for targeted photochemotherapy of breast cancer cells

**DOI:** 10.1038/srep46688

**Published:** 2017-04-21

**Authors:** Yu-Hsiang Lee, Da-Sheng Chang

**Affiliations:** 1 Department of Biomedical Sciences and Engineering, National Central University, Taoyuan City, Taiwan R.O.C; 2 Department of Chemical and Materials Engineering, National Central University, Taoyuan City, Taiwan R.O.C

## Abstract

In this study, we aimed to develop anti-human epidermal growth factor receptor 2 (HER2) indocyanine green (ICG)-doxorubicin (DOX)-encapsulated polyethylene glycol-poly(lactic-co-glycolic acid) diblock copolymeric nanoparticles (HIDPPNPs) to explore the co-administration of phototherapy and chemotherapy for HER2-overexpressing breast cancer, a highly aggressive and medicine-resistant breast carcinoma. The HIDPPNPs were fabricated using a solvent evaporation technique followed by carbodiimide-mediated antibody conjugation on the nanoparticle surface. Compared with freely dissolved ICG, the HIDPPNPs conferred enhanced thermal stability to the entrapped ICG, were able to generate a hyperthermia effect at concentrations ≥1 μM ICG equivalent and provided increased production of singlet oxygen under 808-nm laser irradiation with an intensity of 6 W/cm^2^. Furthermore, the uptake efficiency of the HIDPPNPs in MDA-MB-453/HER2(+) cells was approximately 2-fold higher than that in MCF7/HER2(−) cells, demonstrating that the HIDPPNPs specifically target HER2-expressing cells. Based on the viability analysis, the HIDPPNPs exhibited effective cytotoxicity upon light exposure (808 nm; 6 W/cm^2^), and the resulting cell death rate was even higher than that caused by using twice amount of encapsulated DOX or ICG alone. These results indicate that the developed HIDPPNPs may serve as a feasible tool for use in anti-HER2 breast cancer therapy with reduced chemotoxicity.

According to the statistics of the World Health Organization, breast cancer is the most frequently diagnosed cancer and the leading cause of cancer death among females worldwide^1^. Although breast cancer therapies have continuously advanced over the last few decades, metastatic breast cancer remains incurable, and the 5-year overall survival rate is still <25%^2^, indicating that an effective therapeutic strategy is still urgently needed. Among the various types of breast cancer, human epidermal growth factor receptor 2 (HER2)-overexpressing breast cancer, which accounts for nearly 30% of breast cancers located in either primary tumours or metastatic sites^3^, is known to have a poorer prognosis^4^,^5^ and to respond poorly to chemotherapy and/or hormonal therapy^6^. Moreover, HER2-overexpressing breast cancer is known to be more aggressive and resistant to medicinal treatment^7^,^8^, suggesting that improving the method of tumour destruction rather than persistently changing the anticancer drugs used may be required to truly cure this type of breast cancer.

Doxorubicin (DOX) is a commonly used anticancer drug that has received US FDA approval for a wide spectrum of neoplastic diseases, and the mechanisms of its antitumour effects have been known to originate from DNA intercalation and free radical generation^9^. However, the use of DOX also carries various drawbacks, such as a lack of tumour specificity, dose-dependent cardiotoxicity, and increased drug resistance, and all of these seriously restrict its clinical application^9^. To circumvent these challenges, the co-administration of anticancer agents or therapeutics is often considered a potential strategy for cancer treatment since it may help to decrease the dose of each drug used and thus reduce the dose-dependent toxicity of the drug in non-target normal cells/tissues, leading to an improved clinical outcome and reduced side effects compared with those resulting from the administration of a single drug. Among various anticancer therapies, noninvasive near-infrared (NIR)-based phototherapy has gained increasing attention as an adjuvant to breast cancer chemotherapy since it enables (1) increased tissue penetration efficacy compared to that using visible light, (2) enhanced membrane permeability for drug uptake, and (3) moderate toxicity to normal cells/tissues through the use of targeted photosensitive agents and/or spatially controlled light irradiation^10^,^11^. Generally, phototherapy is carried out by hyperthermia and/or reactive oxygen species (ROS) generated from photosensitizers under light illumination in the presence of oxygen; the former may cause thermal ablation of cancer cells (i.e., photothermal therapy (PTT)), while the latter may seriously interfere cellular metabolism and thus trigger programmed cell death (i.e., photodynamic therapy (PDT))^11^,^12^,^13^. No matter which mechanism is employed, the photosensitizer plays a key role in the effectiveness of phototherapy.

Indocyanine green (ICG) is a US FDA-approved tricarbocyanine dye that can be absorbed and fluoresce in the region of 650–850 nm. Thus far, in addition to serving as a fluorophore agent for use in diagnostic methodologies such as NIR image-guided oncologic surgery^14^ and fluorescence angiography^15^, ICG is also exploited for cancerous phototherapy that includes breast, brain, and skin tumours^16^,^17^,^18^ due to its capability of heat and singlet oxygen generation upon NIR exposure. However, the drawbacks of using ICG, such as high aqueous degradability^19^ and rapid plasma clearance^20^, detrimentally affect its applicability in the clinic.

Nanomedicine may offer a feasible means for the co-administration of anticancer agents, including ICG, without any of the aforementioned drawbacks because it may provide the advantages of enhanced bioavailability, improved stability, and security to the payload^21^. In this study, we sought to fabricate a type of anti-HER2 ICG-DOX-encapsulated polyethylene glycol (PEG)-poly(lactic-co-glycolic acid) (PLGA) diblock (PEG-b-PLGA) copolymeric nanoparticles (HIDPPNPs) to explore the co-administration of photochemotherapy and target-specific treatment for HER2-overexpressing breast cancer cells. PLGA is the copolymer of poly(lactic acid) and poly(glycolic acid) and is one of the best-defined biomaterials with FDA approval for drug encapsulation due to its biocompatibility, biodegradability, and controllability in terms of drug release^22^. PEG, another FDA-approved polymer, is frequently used to provide reduced toxicity and decrease immunogenicity of the drug carriers^23^. We anticipate that the developed HIDPPNPs may be able to (1) potentially protect the entrapped ICG from degradation caused by external stimuli such as light, heat, and/or pH^19^,^24^, (2) specifically locate the therapeutic region to reduce any off-target cytotoxicity generated by DOX, and (3) provide improved overall anticancer treatment efficacy through the provision of multiple therapeutics that are highly advantageous for tumour destruction. In this study, we first introduce the fabrication of HIDPPNPs in detail, followed by the stepwise investigation of their physicochemical properties, functionalities, and cytotoxicity. To clarify the effects of the HIDPPNPs, two similar products, ICG-DOX-loaded PEG-b-PLGA copolymeric nanoparticles (IDPPNPs) and anti-HER2 ICG-encapsulated PEG-b-PLGA nanoparticles (HIPPNPs), were manufactured and employed as comparisons, depending upon the experimental requirements.

## Results and Discussion

### Analysis of synthetic PEG-b-PLGA copolymer

**Figure 1:**
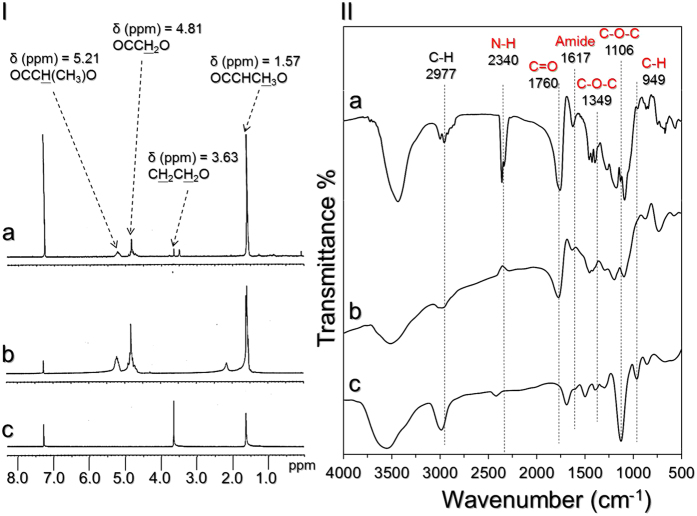
Verification of the synthetic PEG-b-PLGA copolymer. (**I**) The ^1^H NMR spectra of synthetic PEG-b-PLGA (a), acid-terminated PLGA (b), and heterobifunctional PEG (c). (**II**) FTIR transmittance spectra of synthetic PEG-b-PLGA (a), acid-terminated PLGA (b), and heterobifunctional PEG (c) in the wavenumber range of 500–4000 cm^−1^.

The successful conjugation of PEG and PLGA polymers was verified by analysing the characteristic peaks of the formed product (i.e., PEG-b-PLGA copolymer), acid-terminated PLGA, and heterobifunctional PEG using both ^1^H NMR and FTIR spectroscopy. As shown in Fig. 1-I, we found that the NMR spectrum of the synthetic sample (Fig. 1-I(a)) simultaneously shows both characteristic peaks of PLGA and PEG. More specifically, the peaks at δ (ppm) = 1.57 (−CH_3_−), δ (ppm) = 4.81 (−CH_2_−), and δ (ppm) = 5.21 ppm (−CH−) represent the PLGA structure (Fig. 1-I(b)), while the peak at δ (ppm) = 3.63 (−CH_2_CH_2_−) denotes the presence of the PEG structure (Fig. 1-I(c)) in the synthesized product.

According to the FTIR analyses shown in Fig. 1-II, a strong transmittance peak at 1760 cm^−1^ corresponding to the carbonyl stretching frequency of PLGA can be observed in both the synthetic product (Fig. 1-II(a)) and the acid-terminated PLGA (Fig. 1-II(b)). The three transmittance peaks at 949, 1106, and 1349 cm^−1^, which represent -CH out-of-plane bending vibration, C-O-C stretching, and asymmetric stretching, respectively, appear in both the spectra of the synthesized sample (Fig. 1-II(a)) and of the heterobifunctional PEG (Fig. 1-II(c)), indicating the presence of the PEG structure in the synthetic product^25^,^26^. Moreover, in the FTIR spectrum of the synthesized compound, the peak at 2340 cm^−1^ is attributed to N-H stretching of the amino compounds, and a small but well-resolved peak at 1617 cm^−1^ represents the hydrogen-bonded carbonyl of the amide, indicating that a carbonyl amide linkage (CONH) was successfully generated through a carbodiimide-mediated crosslinking reaction. Overall, these results confirm that the synthesis of the PEG-b-PLGA copolymer was successfully carried out.

### Verification of anti-HER2-mAb conjugation on the nanoparticle surface

**Figure 2:**
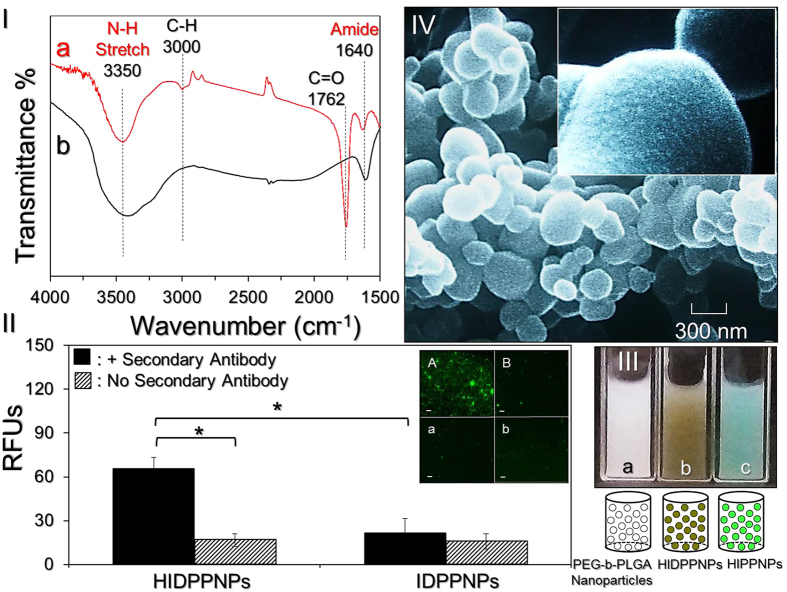
Assessment of the physicochemical properties of HIDPPNPs. (**I**) The FTIR transmittance spectra of HIDPPNPs (a) and free anti-HER2-mAb within the wavenumber range of 1500–4000 cm^−1^ (b). (**II**) Verification of the presence and bioactivity of anti-HER2-mAbs on the HIDPPNPs. The inset photographs are the representative fluoromicroscopic images of HIDPPNPs (A/a) and IDPPNPs (B/b) with (A/B) and without (a/b) conjugation of fluorescent anti-mouse IgG secondary antibody at 200X magnification. Scale bar = 10 μm. The intensity of fluorescence expressed in each group was measured by spectrofluorometry at an excitation wavelength of 488 nm and an emission wavelength of 525 nm and was quantitatively represented by RFUs. The values are the mean ± s.d. (n = 3) (*P < 0.05). (**III**) Photographs and schematic diagrams of blank PEG-b-PLGA nanoparticles (a), HIDPPNPs (b), and anti-HER2 ICG-loaded PEG-b-PLGA nanoparticles (HIPPNPs) (c). (**IV**) SEM images of the HIDPPNPs at magnifications of 15,000X and 60,000X (inset).

Figure 2-I shows the FTIR spectra of the HIDPPNPs and anti-HER2-mAb. The results show that the peaks at 1762 and 3000 cm^−1^ denoting C=O and C-H, respectively, are only present in the HIDPPNP sample (Fig. 2-I(a)) and that they are attributable to the compositions of PEG and/or PLGA. In addition, both groups exhibited peaks at 1640 and 3350 cm^−1^ that represent amide I bending and N-H stretching, respectively, and these outcomes clearly identify the presence of the antibody on the nanoparticle surface.

Figure 2-II shows the secondary antibody assay results. Our data show that the HIDPPNPs displayed remarkable fluorescent expression after treatment with the secondary antibody (Fig. 2-II; inset image A) and that the level of fluorescence was four-fold (*P* = 0.008) higher than that obtained from the HIDPPNPs without the secondary antibody (Fig. 2-II, inset image a). In contrast, the fluorescence levels in the IDPPNPs with and without treatment with the secondary antibody are insignificantly different (Fig. 2-II; inset image B and b; *P* = NS), and their expression levels are similar to those obtained from the HIDPPNPs without the secondary antibody (Fig. 2-II; inset image a, *P* = NS). These results clearly show that the HIDPPNPs, rather than the IDPPNPs, have an affinity to the secondary antibody, indicating that the anti-HER2-mAbs were definitely bound on the surface of the HIDPPNPs and were able to provide intact bioconjugation activity after the carbodiimide-mediated crosslinking reactions. The conjugation efficiency of anti-HER2-mAb on the HIDPPNPs was approximately 12 ± 0.8 wt% according to BCA-mediated protein analysis.

### Characterization of HIDPPNPs

Figure 2-III(a–c) shows photographs of the blank PEG-b-PLGA nanoparticles, HIDPPNPs, and anti-HER2 ICG-encapsulated PEG-b-PLGA nanoparticles (HIPPNPs). The brown emulsified appearance of the HIDPPNPs (Fig. 2-III,b) clearly illustrates the configuration of their ICG and DOX payloads compared with those of the other two samples. According to the SEM observations (Fig. 2-IV), the HIDPPNPs exhibited a particulate morphology with a rough surface (Fig. 2-IV; inset image). The mean size of the HIDPPNPs was 266 ± 4.26 nm with a polydispersity index of 0.07–0.12 after filtration, and the surface charge was approximately −12 ± 4.48 mV according to the DLS measurement. Equation (1) and Eq. (2) were used to calculate the encapsulation efficiencies of ICG and DOX in the fabricated HIDPPNPs, at 80 ± 3.3% and 37 ± 5%, respectively, and these values are comparable to previously reported results^27^. Furthermore, the loading rates of ICG and DOX in the HIDPPNPs were approximately 0.5 ± 0.08 wt% and 0.2 ± 0.07 wt%, respectively.

### Thermal stability of HIDPPNP-entrapped ICG and efficiency of DOX release

**Figure 3:**
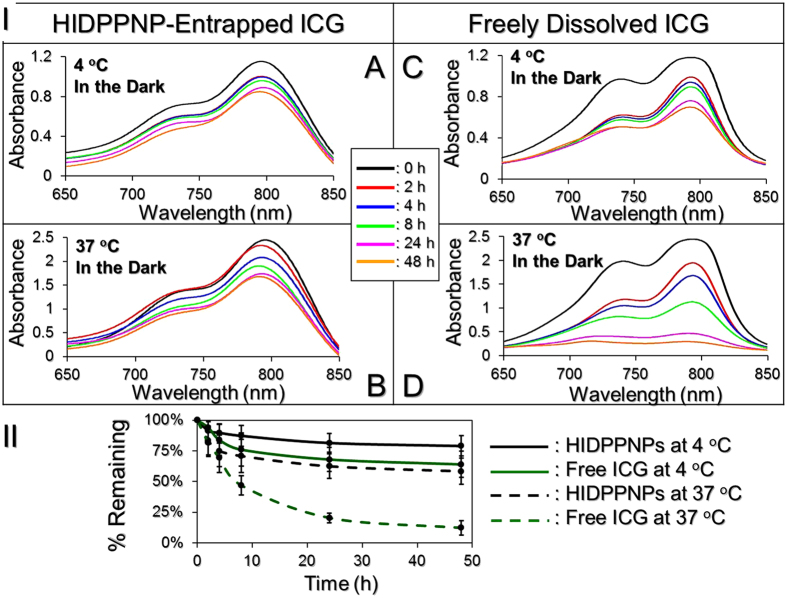
Assessment of the thermal stability of HIDPPNP-entrapped ICG and freely dissolved ICG in PBS. (**I**) A–D show the UV-Vis spectra of HIDPPNPs (A and B) and freely dissolved ICG (C and D) in PBS under 4 (A and C) or 37 °C (B and D) incubation without light illumination for 0, 2, 4, 8, 24, and 48 h. The absorbance at λ = 780 nm in each spectrum denotes the level of ICG for those specific conditions. (**II**) Variations in the ICG amount in the polymer matrix (i.e., HIDPPNP) or PBS solution under 4 and 37 °C environments within 48 h. The values are the mean ± s.d. (n = 3).

Figure 3-I presents the degradation profiles of the HIDPPNP-entrapped ICG (Fig. 3-I,A and B) and freely dissolved ICG in PBS (Fig. 3-I,C and D) under incubation at 4 or 37 °C in the dark for 48 h. After analysis of the absorbance at λ = 780 nm for each set (Fig. 3-II), we found that the percentages of ICG remaining in the polymer matrix were 1.5- and 4.8-fold higher than those remaining in PBS samples after 48 h at 4 and 37 °C, respectively, indicating that the thermal degradation of ICG was dramatically reduced after entrapment in the HIDPPNPs. Moreover, the *K*_d_ analyses (Table 1) indicated that the anti-degradability of the HIDPPNP-entrapped ICG at 4 and 37 °C was significantly enhanced by 2 (*P* = 0.0176)- and 4 (*P* = 0.0043)-fold, respectively, compared to the free ICG with equal treatment for 48 h. These outcomes clearly demonstrate that the thermal stability of ICG can be markedly improved after entrapment in the HIDPPNPs.

**Figure 4:**
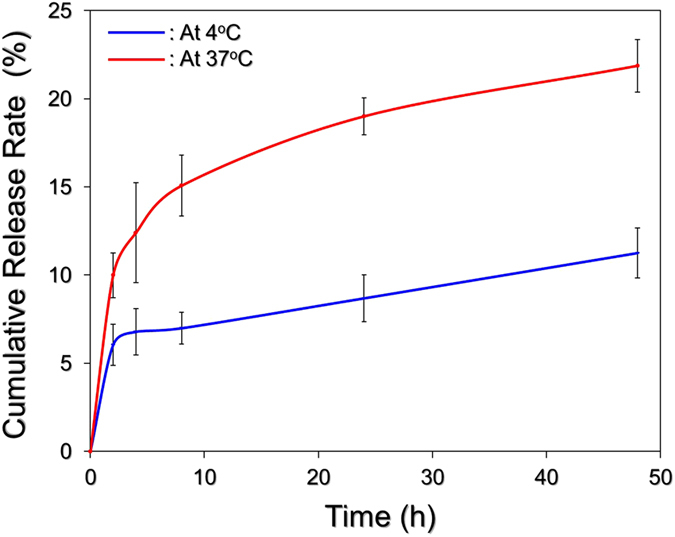
Kinetic release profiles of DOX from HIDPPNPs *in vitro*. The cumulative release curves of DOX at 4 °C (blue) and 37 °C (red) were established by measuring the concentrations of DOX in the supernatant through UV-Vis spectrometry (λ = 485 nm) after incubation at each temperature setting for 0, 2, 4, 8, 24, and 48 h. The values are the mean ± s.d. (n = 3).

The cumulative percentages of DOX released from the HIDPPNPs at 4 and 37 °C were concurrently measured, and the results are shown in Fig. 4. Both groups exhibited a two-phased DOX release profile, and the overall release rates after 48-h maintenance at 4 and 37 °C were 11.2 ± 1.4% and 21.8 ± 1.5%, respectively. Such a biphasic drug release profile, consisting of an initial burst release followed by a sustained slow release of DOX, is in agreement with the previously reported release profile of DOX from PLGA^28^,^29^. The burst release is due to the release of any drug molecules that are located close to the nanoparticle surface and may occur quickly, while sustained release occurs as a result of the subsequent diffusion of drug molecules in the polymer matrix and/or degradation of the nanoparticles. Moreover, in comparison to the similar products reported previously, the release rate of DOX from HIDPPNPs is lower than that obtained from DOX-loaded PLGA nanoparticles^29^ or ICG-DOX-loaded lipid-PLGA nanoparticles^27^, implicating that the DOX is relatively stable in the HIDPPNPs. We speculate that the reduced release efficiency of DOX from the HIDPPNPs was caused by (1) a higher degree of steric hindrance on the particle surface due to tangled PEG moieties and/or antibodies and (2) improved stability of the nanoparticles because the hydrophilic PEG may act as a barrier to reduce interactions with foreign molecules by steric and/or hydrated repulsion^30^, thereby providing enhanced shelf stability to the HIDPPNPs in the aqueous solution.

### Target specificity of HIDPPNPs

**Figure 5:**
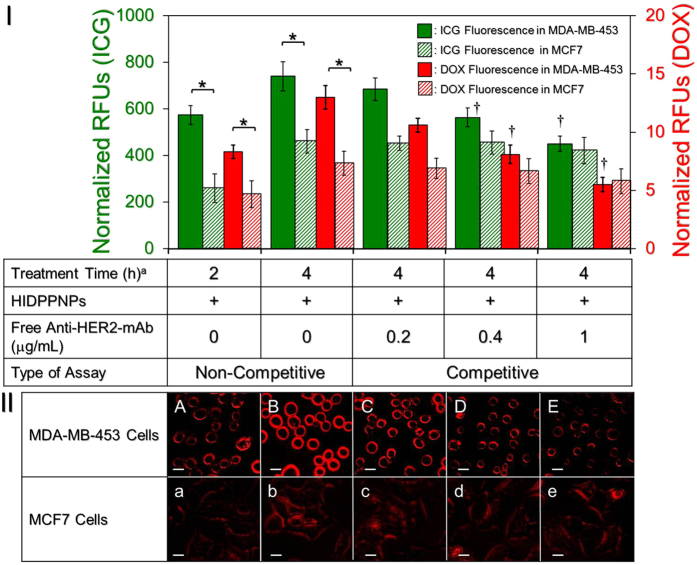
Assessment of the HER2-target specificity of HIDPPNPs. (**I**) The upper panel shows the levels of fluorescence expressed from the MCF7 and MDA-MB-453 cells. For the non-competitive assay, each type of cell was treated with HIDPPNPs with 1-μM ICG/0.8-μM DOX equivalent concentration for 2 and 4 h, respectively. For the competitive assay, each type of cell was separately pre-treated with 0.2-, 0.4-, or 1-μg/mL free anti-HER2-mAb for 2 h followed by co-culture with the HIDPPNPs at 37 °C for 4 h. The intensities of the fluorescence were measured using spectrofluorometry performed at excitation and emission wavelengths of 750 and 838 nm, respectively, for the detection of ICG and at excitation and emission wavelengths of 485 and 590 nm, respectively, for the detection of DOX immediately after the HIDPPNPs were removed. The intensities were quantitatively represented by RFUs after normalization to the control signal. The values are the mean ± s.d. (n = 3). ^a)^Treatment time denotes the incubation time that cells were co-cultured with the HIDPPNPs. ^†^*P* < 0.05 as compared to the group co-cultured with the HIDPPNPs for 4 h in the absence of free anti-HER2-mAb. *P < 0.05. (**II**) The bottom panel shows the DOX-derived fluoromicrography images of MDA-MB-453 (A–E) and MCF7 (a–e) cells after co-cultured with the HIDPPNPs for 2 (A and a) and 4 (B/b–E/e) h without (A/a and B/b) and with pre-treatment of 0.2- (C and c), 0.4- (D and d), or 1- (E and e) μg/mL free anti-HER2-mAb. All images were photographed at 200X magnification. Scale bar = 10 μm.

Figure 5-I shows the levels of ICG- and DOX-induced fluorescence expressed from the MDA-MB-453 (HER2(+)) and MCF7 (HER2(−)) cells after treatment with HIDPPNPs for 2 and 4 h with and without antibody competition. In terms of the non-competitive study, the analysis of the fluorescent intensities showed that compared to those obtained from the MCF7 cells, the normalized RFUs detected from the MDA-MB-453 cells were 2.2 (*P* = 0.0425)- and 1.6 (*P* = 0.0423)-fold higher based on the ICG-derived RFUs, and 2 (*P* = 0.0169)- and 1.8 (*P* = 0.0355)-fold higher based on the DOX-derived RFUs after treatment with the HIDPPNPs for 2 and 4 h, respectively. These data were consistent with the fluorescence microscopic results (Fig. 5-II), indicating that the density of the HIDPPNPs on the membrane of the MDA-MB-453 cells was remarkably higher than that on the surface of the MCF7 cells (Fig. 5-II; A vs. a, B vs. b) and that the level of fluorescence was positively correlated with the HIDPPNP treatment time (Fig. 5-II; A vs. B. a vs. b). To investigate the mechanism of HIDPPNP uptake for each type of cell, the binding efficiencies of the HIDPPNP on both MDA-MB-453 and MCF7 cells in the presence of competitive anti-HER2-mAb were further examined. As presented in Fig. 5-I, the results show that the ICG-derived RFU significantly decreased 40% (*P* = 0.0283) in MDA-MB-453 cells while it was maintained at a similar level in MCF7 cells (*P* = 0.465) when the dose of free anti-HER2-mAb was increased by five folds (from 0.2 to 1 μg/mL). Similar results can be obtained from detection of the DOX-derived fluorescence using spectrofluorometry (Fig. 5-I) and fluorescent microscopy (Fig. 5-II; C/c–E/e). These outcomes clearly show that the uptake efficiency of HIDPPNP in HER2-expressing cells was markedly affected by the competitive HER2 antibody, manifesting that the internalization of the HIDPPNPs in HER2(+) MDA-MB-453 cells was mainly attributed to HER2 receptor-mediated endocytosis, while that in HER2(−) MCF7 cells was conducted through adsorptive endocytosis since it has been demonstrated as an efficient means for cancer cells to engulf negatively charged PLGA nanoparticles^31^. By comparing the endocytic pathways utilized by the two types of cells, receptor-mediated endocytosis is more efficient and specific than adsorptive endocytosis^32^; thus, the uptake efficiency of the HIDPPNPs in the MDA-MB-453 cells was higher than that in the MCF7 cells. Overall, these results clearly show that the HIDPPNPs are more efficiently internalized/adsorbed by MDA-MB-453/HER2(+) cells; this finding demonstrates the targeted specificity of HIDPPNPs to HER2(+) cells.

### Photodynamic and hyperthermia effects of HIDPPNPs

**Figure 6:**
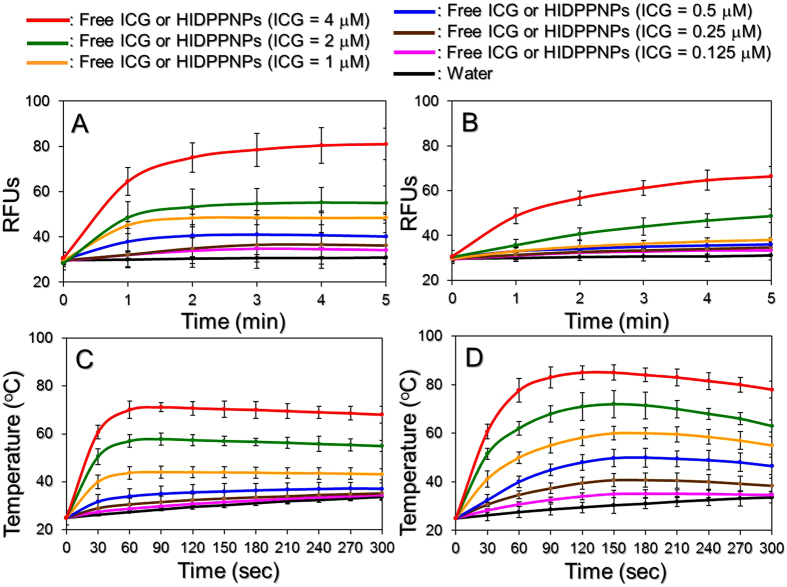
Production of singlet oxygen and the hyperthermia effect of HIDPPNPs under NIR laser irradiation. Upon exposure to an 808-nm laser with an intensity of 6 W/cm^2^, the production of singlet oxygen (**A** and **B**) and the variation in temperature (**C** and **D**) generated from the HIDPPNPs (**A** and **C**) and freely dissolved ICG (**B** and **D**) with concentrations of 0 (water only), 0.125, 0.25, 0.5, 1, 2, and 4 μM ICG equivalent were measured every 60 (for yield of singlet oxygen) or 30 (for variation of temperature) sec for 5 min. The quantity of singlet oxygen was analysed based on the intensity of SOSG-induced fluorescence, which was measured by using spectrofluorometry with excitation and emission wavelengths of 488 and 525 nm, respectively. The temperature at each time point was measured using a digital thermometer. The values are the mean ± s.d. (n = 3) in all examinations.

Figure 6(A and B) shows the profiles of singlet oxygen accumulation as quantitatively represented by RFUs for various concentrations of HIDPPNPs (Fig. 6A) and freely dissolved ICG (Fig. 6B) upon 808-nm laser irradiation with an intensity of 6 W/cm^2^ for 5 min. Our data show that the RFUs for all the groups increased after treatment with NIR irradiation and that the values detected from the HIDPPNPs were higher than those obtained from the same concentrations of ICG solutions throughout the time course. According to the RFU analysis, the HIDPPNPs were able to provide 30% more singlet oxygen within 5 min of NIR laser treatment compared to the freely dissolved ICG under equal ICG concentrations. These results implicate that the HIDPPNPs were able to provide an enhanced photodynamic effect compared to the same concentration of free ICG upon NIR laser exposure.

Figure 6(C and D) shows the profiles of the hyperthermia effect generated from the various concentrations of HIDPPNPs (Fig. 6C) and freely dissolved ICG (Fig. 6D) upon NIR laser irradiation as described above. Our data show that the temperature in each HIDPPNP concentration rapidly increased during the first minute of NIR laser irradiation and was sustained at approximately the same level for the rest of the experiment, resulting in increases of 8.6, 9.5, 10.2, 12.1, 18.2, 30, and 43 °C after 5 min of NIR exposure for the HIDPPNPs with 0 (water only), 0.125, 0.25, 0.5, 1, 2, and 4 μM ICG equivalent concentrations, respectively. In terms of the settings of free ICG, their temperatures quickly increased during the first 60–90 sec of NIR laser irradiation and declined thereafter, yielding increases of 9.6, 13.4, 21.5, 30, 38, and 53 °C after 5 min of NIR exposure for ICG solutions with concentrations of 0.125, 0.25, 0.5, 1, 2, and 4 μM, respectively. These results indicate that the HIDPPNPs were certainly able to generate a photothermal effect upon NIR laser irradiation, although the level of the temperature increase was lower than that achieved with free ICG under the same treatment.

Based on the results shown in Fig. 6, one may question why the HIDPPNPs provided an enhanced photodynamic effect but a relatively lower hyperthermia effect compared to the free ICG. Our explanations are as follows: in contrast with the ICG solution where all the ICG molecules reacted upon NIR laser irradiation, the hyperthermia effect provided by the HIDPPNPs was produced by partially released ICG; therefore, the magnitude of the temperature elevation was moderate compared to that attained with the free ICG. However, since the degradation of ICG in an aqueous medium can be accelerated by light and/or heat^19^, it was expected that the free ICG disintegrated while undergoing the NIR exposure and that such disintegration is unfavourable for the generation of singlet oxygen. This hypothesized simultaneous degradation of free ICG can also be used to explain why the temperature declined after ~150 sec of NIR laser irradiation, as shown in Fig. 6D. Conversely, since the HIDPPNP-entrapped ICG was mostly protected from light illumination, causing its thermal stability to greatly improve as shown in Table 1, we speculate that the singlet oxygen in the HIDPPNP groups was able to be effectively generated via the continuous release of the intact ICG molecules, resulting in an efficient accumulation of singlet oxygen compared to that yielded by free ICG.

**Figure 7:**
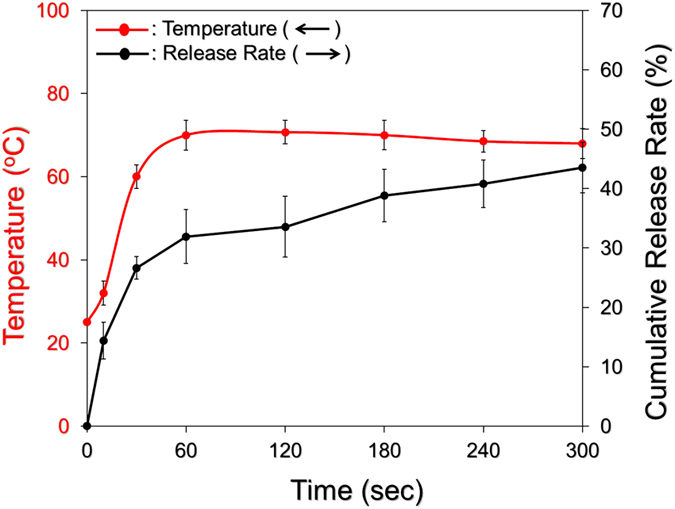
Kinetic release profile of DOX from HIDPPNPs under light exposure *in vitro*. The cumulative release curve of DOX under NIR light irradiation was established by measuring the concentrations of DOX in the supernatant through UV-Vis spectrometry (λ = 485 nm) after operating for 0, 10, 30, 60, 120, 180, 240, and 300 sec. The light illumination was performed using an 808-nm laser with an intensity of 6 W/cm^2^. The red curve indicates the temperature of the medium at each time point when the concentration of released DOX was detected. The values are the mean ± s.d. (n = 3).

The temperature level has been known to play a key role in the efficacy of photothermal therapy. According to previously reported cellular assay results, irreversible cell damage can be obtained after heating at 40–45 °C for 30–60 min^33^, while only 4–6 min is sufficient at 50–52 °C^34^. At temperatures >60 °C, the time required to cause irreversible cell damage decreases exponentially because the denaturation of cytoplasmic proteins and/or enzymes occurs rapidly and may lead to immediate necrosis^35^. Although higher temperatures may provide more opportunities to eradicate tumour cells, operating temperatures in the range of 41–43 °C are frequently utilized in the clinic to minimize their potential deleterious influence on the surrounding normal cells^36^. Based on the results shown in Fig. 7-II, we reason that HIDPPNPs with concentrations ≥1 μM ICG equivalent were able to provide both photodynamic and hyperthermia (T > 42 °C) effects for tumour destruction, whereas the phototherapeutic effect of HIDPPNPs with concentrations ≤0.5 μM ICG equivalent were majorly dependent on the photodynamic efficacy under 808-nm laser irradiation with an intensity of 6 W/cm^2^ for 5 min.

### Efficiency of DOX release under NIR laser irradiation

We subsequently examined the efficiency of DOX release under NIR laser irradiation (808 nm; 6 W/cm^2^), and the result is shown in Fig. 7. Through the use of HIDPPNPs containing 4 μM ICG equivalent, it was observed that the NIR-treated HIDPPNPs also exhibited a two-phased DOX release profile as they were performed without light illumination (Fig. 4), and the cumulative release rate after a 5-min operation was 43.5 ± 4.2%. Given that the bulk temperature (Fig. 7, red curve) was able to reach 70 °C within 60 sec of NIR laser irradiation, which was higher than the *T*_g_ of the acid-terminated PLGA (42–46 °C) used in this study, it is reasonable to conclude that the disintegration of the HIDPPNPs was triggered immediately after irradiation with the NIR laser and led to a burst release of DOX in the first minute of NIR treatment, as indicated in Fig. 7. In comparison with the outcomes shown in Fig. 4, these data clearly indicate that the efficiency of DOX release from the HIDPPNPs was enhanced by NIR laser treatment (808 nm; 6 W/cm^2^).

### *In vitro* cytotoxicity of HIDPPNPs

**Figure 8:**
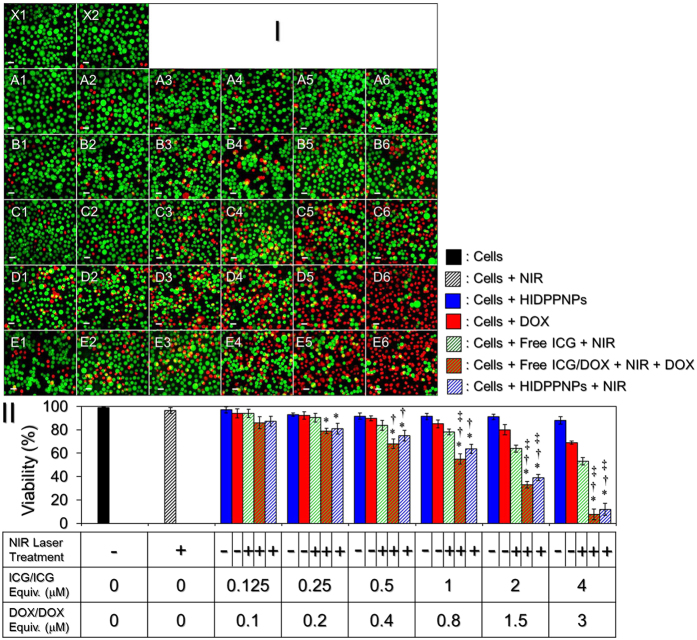
Cytotoxicity of HIDPPNPs to HER2(+) breast cancer cells *in vitro*. (**I**) Representative photomicrographic images of MDA-MB-453 cells under various treatments. Rows A and B represent the groups of cells that were co-cultured with the HIDPPNPs (A) or DOX (B) for 24 h in which the concentrations of DOX/DOX included were set at 0.1 (A1 and B1), 0.2 (A2 and B2), 0.4 (A3 and B3), 0.8 (A4 and B4), 1.5 (A5 and B5), and 3 (A6 and B6) μM. Row C represents the group of cells precocultured with 0.125 (C1), 0.25 (C2), 0.5 (C3), 1 (C4), 2 (C5), or 4 (C6) μM of free ICG for 4 h, then exposed to NIR laser irradiation for 5 min followed by incubation at 37 °C for an additional 24 h. Rows D and E represent the groups of cells that were precocultured with combination of free ICG and DOX (D) or HIDPPNPs (E) for 4 h in which the concentrations of ICG and DOX (ICG/DOX) were set at 0.125/0.1 (D1 and E1), 0.25/0.2 (D2 and E2), 0.5/0.4 (D3 and E3), 1/0.8 (D4 and E4), 2/1.5 (D5 and E5), and 4/3 (D6 and E6) μM, then exposed to NIR laser irradiation for 5 min followed by incubation at 37 °C in the presence (D) or absence (E) of DOX with originally designated concentration for an additional 24 h. X1 is the blank group in which the cells were treated with neither chemicals (ICG and DOX) nor laser exposure. X2 denotes the cells treated with NIR laser irradiation for 5 min followed by incubation at 37 °C for 24 h. The green and red cells stained by calcein-AM and PI represent live and dead cells, respectively. All images were photographed using a fluorescence microscope at 200X magnification. Scale bar = 30 μm. (**II**) Quantitative analyses of the viabilities of MDA-MB-453 cells after treatment with DOX, free ICG, or HIDPPNPs in various doses and/or handling methods as indicated in the figure. NIR laser irradiation was performed by an 808-nm laser with an intensity of 6 W/cm^2^ for 5 min. The cellular viability was determined by using haemocytometer with a trypan blue exclusion method. The values are the mean ± s.d. (n = 3). *P < 0.05 compared to the group treated with HIDPPNPs without light illumination. ^†^*P* < 0.05 compared to the group treated with an equal concentration of DOX alone. ^‡^*P* < 0.05 compared to the group treated with an equal concentration of free ICG and NIR laser irradiation.

Figure 8 shows the viabilities of MDA-MB-453 cells after treatment with various doses of ICG, DOX, or HIDPPNPs with and without NIR laser irradiation (808 nm, 6 W/cm^2^; 5 min) as described above. The concentrations of the free ICG and DOX examined in the cytotoxicity experiment corresponded to the doses that can be provided from the HIDPPNPs. From the haemocytometer analyses (Fig. 8-II), our data show that the viability of the cells treated with NIR laser irradiation in the absence of an agent (Fig. 8-I; X2) was 96.5%, indicating that the slight temperature increase due to the NIR laser irradiation (Fig. 6) was nontoxic to the cells. In contrast, a dose-dependent increase in cytotoxicity was observed in the agent-treated cells (Fig. 8-I; rows A–E), in which the cells treated with HIDPPNPs and NIR laser irradiation (Fig. 8-I,E1–E6) underwent higher levels of cell death as compared to the cells treated with HIDPPNPs without light illumination (Fig. 8-I,A1–A6), those treated with DOX alone (Fig. 8-I,B1–B6), and those treated with free ICG + NIR laser irradiation (Fig. 8-I,C1–C6), whereas the level of cell death was similar with that obtained from the cells with free ICG/DOX-mediated photochemotherapy (Fig. 8-I,D1–D6). Based on the finding that the viability of the cells treated with HIDPPNPs and NIR laser irradiation was lower than that of the cells without NIR exposure throughout the dose range, the HIDPPNPs were characterized as slightly toxic to the cells in the absence of light illumination. Moreover, we found that the mortality of the cells treated with HIDPPNPs and NIR laser irradiation increased exponentially when the concentrations of ICG and DOX provided by the HIDPPNPs were ≥ 1 and 0.8 μM, respectively (Fig. 8-II). As shown in Fig. 8-II, viabilities of 64%, 40%, and 12% were obtained for the cells with 1, 2, and 4 μM ICG in the HIDPPNPs, respectively, and those results were 1.2 (*P* = 0.0372)-, 1.6 (*P* = 0.0162)-, and 4.4 (*P* = 0.0173)-fold lower than the viability values obtained for the groups with equal concentrations of free ICG. Furthermore, those results were 1.3 (*P* = 0.0011)-, 2.1 (*P* = 0.0004)-, and 5.8 (*P* = 0.0022)-fold lower than the viability values obtained for the groups with equal doses of DOX alone (0.8, 1.5, and 3 μM DOX), respectively. Based on the results shown in Fig. 6, we reason that the significant cytotoxicity of HIDPPNPs with concentrations ≥1 μM ICG equivalent was achieved due to the relatively high dose of DOX and the combination of the photodynamic and photothermal effects, while the HIDPPNPs with concentrations ≤0.5 μM ICG equivalent only provided a mild photodynamic effect and a relatively low dose of DOX and thus resulted in a moderate efficacy of cytotoxicity.

In this study, the HIDPPNP-mediated cancer cell killing was performed by 5 min of phototherapy after 4-h uptake of the photosensitizers (i.e., HIDPPNPs) followed by 24 h of chemotherapy. To minimize the chemotherapy-induced side effects, the HIDPPNPs were used with up to 3 μM of DOX, which is lower than the clinical dose of DOX (typically ≥ 10 μM)^37^. However, robust cytotoxicity of the HIDPPNPs upon NIR exposure was still obtained in each setting, and the resulting mortality was even higher than that caused by using twice amount of encapsulated DOX alone (Fig. 8-II), indicating that the phototherapy played a crucial role in the HIDPPNP-mediated anticancer treatment. The importance of phototherapy in such combined therapeutics can also be identified through comparing the viability of cells treated by free DOX alone (Fig. 8-I; Row B) to that treated with both free ICG and DOX (Fig. 8-I; Row D). In this study, although the effect of photochemotherapy generated from the HIDPPNPs *in vitro* can be achieved by using free ICG and DOX individually (Fig. 8-I; Row D vs. E), it is predictable that the latter may not be an appropriate approach for use in the clinic due to defects of applicability such as lack of target-ability, insufficient stability, and rapid plasma clearance for the free ICG molecule as described above. On the contrary, the HIDPPNP seemed to be a more beneficial phototoxic agent than free ICG according to its physicochemical properties. Based on the *K*_d_ analysis as shown in Table 1, the degradation efficiency of free ICG within 4 h at 37 °C was approximately four-fold higher than that of the HIDPPNP-entrapped ICG, implicating that the amount of intact ICG transferred by free molecules was lower than that delivered by HIDPPNPs. In addition, the HIDPPNPs can be efficiently internalized by MDA-MB-453 cells due to their HER2-binding specificity (Fig. 5), and the entrapped ICG may be protected from the immediate degradation caused by low pH in the late endosome^24^,^38^ after the HIDPPNP entered into the cell. These properties are anticipated to be able to improve the availability of the HIDPPNPs for use in phototherapy; therefore, the required dose of chemotherapeutics may be reduced without compromising the effect of cancer therapy through the use of HIDPPNPs.

In this study, we successfully fabricated HIDPPNPs for targeted photochemotherapy of HER2(+) breast cancer cells. We not only investigated their physicochemical properties and functionalities but also demonstrated the availability of HIDPPNPs at different dosages for the destruction of HER2-overexpressing MDA-MB-453 breast cancer cells *in vitro*. In addition to the aforementioned merits of HIDPPNPs, the constituent PEG oriented toward the external aqueous phase may allow the nanoparticles to have a prolonged circulation time in the bloodstream due to its lower immunogenicity^23^, and these characteristics are particularly favourable for drug delivery to tumour cells through the enhanced permeability and retention (EPR) effect^39^. Therefore, we anticipate that the HIDPPNPs may be able to provide an improved cancer therapy *in vivo* compared to that provided by free ICG or DOX alone. However, further studies are certainly required and such efforts are currently in progress.

## Methods

### Synthesis of PEG-b-PLGA copolymer

A quantity of 60 mg of acid-terminated PLGA (50:50, *M*_n_ = 7–17 kDa) was first mixed with *N*-(3-dimethylaminopropyl)-*N*’-ethylcarbodiimide hydrochloride (EDC) and *N*-hydroxysuccinimide (NHS) (molar ratio of EDC/NHS = 2:1) in a total of 2 mL of dichloromethane (DCM) under stirring at room temperature in the dark for 2 h. After the mixture was washed three times with ice-cold methanol, the educted ester chemical was mixed with poly(ethylene glycol) 2-aminoethyl ether acetic acid (heterobifunctional PEG; COOH-PEG-NH_2_, *M*_n_ = 3.5 kDa) in a total of 3 mL of DCM (6 mg/mL) under stirring at ambient temperature in the dark for 24 h. The synthetic compound (i.e., PEG-b-PLGA) was precipitated by adding ice-cold methanol. After the compound was washed twice with methanol, the collected product was vacuum-dried and stored at 4 °C until use. The characteristics of the synthesized PEG-b-PLGA were identified using proton nuclear magnetic resonance (^1^H NMR) and Fourier transform infrared (FTIR) techniques.

### Fabrication of HIDPPNPs

The ICG-DOX-loaded PEG-b-PLGA copolymeric nanoparticles (i.e., IDPPNPs) were first prepared using a modified emulsification in association with a solvent evaporation method. Briefly, 1.3 mL of a DMSO/methanol/DCM (v/v/v = 1: 2:10) mixture containing 1 mg of DOX, 1 mg of ICG, and 60 mg of PEG-b-PLGA was added dropwise into 20 mL of polyvinyl alcohol (PVA) solution (0.2%, w/v) and sonicated afterward. The emulsified solution was then stirred for another 4 h under ambient temperature to allow organic solvent evaporation. The IDPPNPs were collected by centrifugation at 20000 × g for 30 min, followed by two washes with PBS. The IDPPNPs were resuspended in 1 mL of PBS and immediately subjected to antibody decoration.

The conjugation of anti-HER2-mAbs on the surface of the IDPPNPs was performed by a carbodiimide-mediated method as reported previously^28^. To reduce the size dispersity of the product, the yielded HIDPPNPs were further filtrated through a 0.45-μm filter. The harvested HIDPPNPs were then lyophilized and stored at 4 °C for further use. The overall procedures of the HIDPPNP fabrication are illustrated in Supplementary Fig. S1.

### Evaluation of effectiveness of antibody conjugation

The presence and bioactivity of anti-HER2-mAbs on the nanoparticle surface were verified by FTIR spectroscopy and a secondary antibody assay through use of a fluorescent anti-mouse immunoglobulin G (IgG) secondary antibody as the probe. The quantity of antibody on the HIDPPNPs was evaluated using a Pierce BCA Protein Assay Kit (Thermo Fisher Scientific, Waltham, MA) according to the manufacturer’s instructions. The antibody conjugation efficiency was represented as the ratio of the weight (μg) of decorated anti-HER2-mAb to the weight (mg) of the nanoparticles examined.

### Evaluation of the physicochemical properties of HIDPPNPs

The size distribution and surface charge of the HIDPPNPs were measured using the dynamic light scattering (DLS) technique. The morphology of the HIDPPNPs was detected using a scanning electron microscope with 20 kV accelerating voltage. The encapsulation efficiency (*E*) of the drug (ICG or DOX) was calculated by the formula:


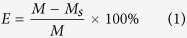


where *M* is the total amount of ICG or DOX used for the HIDPPNP manufacture and *M*_*s*_ denotes the amount of unencapsulated drug molecules remaining in the supernatant. Both *M* and *M*_*s*_ were determined by UV-Vis spectrometry (λ_abs_ = 780 nm for ICG; 485 nm for DOX) according to Beer-Lambert’s law. The loading rate of the drug molecules (ICG or DOX) in the HIDPPNPs (*C*; wt%) was evaluated by the formula:


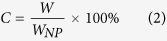


where *W*_NP_ is weight of the HIDPPNPs and *W* denotes the weight of the ICG or DOX encapsulated in the HIDPPNPs examined (~*M* × *E*).

### Analysis of the stability of HIDPPNPs

The thermal stability of the HIDPPNP-entrapped ICG and the release kinetics of entrapped DOX at 4 and 37 °C were measured in this study. All the HIDPPNPs-containing tubes were wrapped in foil to prevent light illumination during the experiment. After treatment for 2, 4, 8, 24, and 48 h, the HIDPPNPs and their supernatant that were collected at both temperature settings were subjected to spectrophotometry at λ_abs_ = 780 (for collected HIDPPNPs) and 485 (for collected supernatant) nm to analyse the amount of ICG remaining in the nanoparticles and the amount of DOX released to the bulk phase, respectively. The cumulative release rate of DOX (*CR*_D_) at each time point was calculated using the formula:


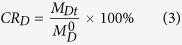


where *M*^0^_D_ represents the amount of DOX originally encapsulated in the HIDPPNPs tested and *M*_Dt_ denotes the amount of DOX detected in the supernatant at a specific time *t* > 0. The ICG freely dissolved in PBS was employed as the control for the study of the thermal stability of HIDPPNP-entrapped ICG. The degradation rate coefficient (*k*_d_) of ICG in each group was determined based on the dynamic method^40^.


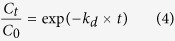


where *C*_0_ and *C*_t_ denote the concentrations of ICG in the HIDPPNPs or PBS (control) at time *t* = 0 and a specific time *t* > 0, respectively.

### Cell culture

The MCF7 cells (HER2(−) human breast adenocarcinoma cell line; ATCC, Manassas, VA) were cultured in Eagle’s minimum essential medium supplemented with 10% foetal bovine serum (FBS), 2 mM L-glutamine, 1.5 g/L sodium bicarbonate, 0.1 mM non-essential amino acids, 1.0 mM sodium pyruvate, 0.01 mg/mL bovine insulin, and 100 U/mL penicillin/streptomycin and maintained in a 37 °C incubator balanced with 5% CO_2_ and 100% humidity. The MDA-MB-453 cells (HER2(+) human breast metastatic carcinoma cell line; ATCC) were cultured in Leibovitz’s L-15 medium supplemented with 10% FBS, 2 mM L-glutamine, and 100 U/mL penicillin-streptomycin and maintained in a 37 °C incubator without CO_2_.

### Examination of the target specificity of HIDPPNPs

The target specificity of the HIDPPNPs was determined by comparing the efficiency of the adsorption of HIDPPNPs in the MDA-MB-453/HER2(+) cells to that in the MCF7/HER2(−) cells. For the non-competitive assay, 1.5 × 10^6^ of each type of cells were aliquoted into three wells of a 24-well culture plate and incubated at 37 °C for 24 h. The HIDPPNPs were added to two of the three wells (1 μM ICG and 0.8 μM DOX equivalents) and then incubated at 37 °C for 2 and 4 h, respectively. The well without HIDPPNPs was employed as the control. In terms of the HER2 competitive inhibition study, free anti-HER2-mAbs with concentration of 0.2, 0.4, and 1 μg/mL were separately pre-incubated with MDA-MB-453 and MCF7 cells for 2 h at 37 °C prior to addition of nanoparticles. After co-cultured with the HIDPPNPs for 4 h, the cells were washed twice with PBS and subjected to nanoparticle adsorption analyses. At each time point, the cells were washed twice with PBS and then detected by fluorescence microscopy. Furthermore, the fluorescent intensities of the cells expressed from the HIDPPNP-entrapped ICG and DOX incorporated were measured by spectrofluorometry performed with excitation/emission wavelengths of 750 /838 nm and 485 /590 nm, respectively. In this study, the intensity of fluorescence was quantitatively represented by relative fluorescence units (RFUs), and the efficiency of cellular uptake of the HIDPPNPs was analysed by the normalized RFUs against the control.

### Measurement of the production of HIDPPNP-induced singlet oxygen

The production of singlet oxygen generated from the HIDPPNPs under 808-nm laser irradiation with an intensity of 6 W/cm^2^ was measured using singlet oxygen sensor green (SOSG; Life Technologies, Carlsbad, CA) as the fluorescent probe according to the manufacturer’s instructions. The level of SOSG-induced fluorescence in each group was detected using a spectrofluorometer with 488 and 525 nm of excitation and emission wavelengths, respectively, every 60 sec for 5 min and was quantitatively represented by RFUs.

### Measurement of the HIDPPNP-induced hyperthermia effect

To evaluate the photothermal effect of the HIDPPNPs, 200 μL of PBS containing HIDPPNPs with 0 (no HIPPNPs), 0.125, 0.25, 0.5, 1, 2, and 4 μM ICG equivalent were separately irradiated by an 808-nm laser with an intensity of 6 W/cm^2^ in one well of a 96-well culture plate. The temperature of each group was recorded every 30 sec for 5 min using a digital thermometer.

### *In vitro* cytotoxicity assay

To evaluate the photochemotherapeutic capacity of the HIDPPNPs, 6.4 mL of culture medium containing 3.2 × 10^6^ MDA-MB-453 cells was aliquoted into 32 wells of a 96-well culture plate and incubated at 37 °C for 24 h. Afterward, freely dissolved ICG or DOX was separately added to six wells, combination of free ICG and DOX was added to six wells, and the HIDPPNPs were added to 12 wells. The concentrations of free ICG and DOX corresponded to their doses provided by the HIDPPNPs and were set as 0.125, 0.25, 0.5, 1, 2, and 4 μM for ICG and 0.1, 0.2, 0.4, 0.8, 1.5, and 3 μM for DOX. After incubation at 37 °C for 4 h, the cells in the six wells with free ICG, six wells with both free ICG and DOX, and another six wells with HIDPPNPs were washed twice with PBS and treated with NIR exposure (808 nm; 6 W/cm^2^) for 5 min. The cells that originally treated with free ICG or HIDPPNPs were then incubated at 37 °C for an additional 24 h and subjected to viability examination. The cells that originally treated with both free ICG and DOX were then incubated at 37 °C in the presence of DOX with originally designated concentration for an additional 24 h followed by viability analysis. For the groups without laser treatment, the viabilities of the cells in the six wells with free DOX and the other six wells with HIDPPNPs were directly measured after a 24-h incubation. The cell viability in each well was assessed using both a haemocytometer and a calcein-AM/propidium iodide (concentration ratio = 2:3) staining approach.

### Statistical analysis

All data were acquired from three independent experiments and are presented as the mean ± standard deviation (s.d.). Statistical analyses were conducted using MedCalc software in which comparisons for one condition between two groups were performed by Student’s *t*-test with a significance level of *P* < 0.05 throughout the study.

## Additional Information

**How to cite this article**: Lee, Y.-H. and Chang, D.-S. Fabrication, characterization, and biological evaluation of anti-HER2 indocyanine green-doxorubicin-encapsulated PEG-b-PLGA copolymeric nanoparticles for targeted photochemotherapy of breast cancer cells. *Sci. Rep.* **7**, 46688; doi: 10.1038/srep46688 (2017).

**Publisher's note:** Springer Nature remains neutral with regard to jurisdictional claims in published maps and institutional affiliations.

## Supplementary Material

Supplementary Information

## Figures and Tables

**Table 1:** Degradation percentages and degradation rate coefficients of the HIDPPNP-entrapped ICG and freely dissolved ICG under various treatments for 48 h.

Group	% ICG degraded	*k*_d_ (h^−1^)
HIDPPNP-entrapped ICG		
4 °C in the dark	21.1%*	0.0049*
37 °C in the dark	41.7%*	0.0112*
Freely dissolved ICG in PBS		
4 °C in the dark	36.8%	0.0093
37 °C in the dark	87.8%	0.0438

*P < 0.05 compared to the group with freely dissolved ICG in PBS under equal heating treatments.
