# Supplementary material for: Fabrication, characterization, and biological evaluation of anti-HER2 indocyanine green-doxorubicin-encapsulated PEG-b-PLGA copolymeric nanoparticles for targeted photochemotherapy of breast cancer cells
Source: Sci Rep. 2017 Apr 21;7:46688. doi: 10.1038/srep46688 (PMC5399361; doi:10.1038/srep46688)
Supplement: Supplementary Information [file srep46688-s1.pdf]

Supplementary Figure

Fabrication, characterization, and biological evaluation of anti-HER2 indocyanine green-doxorubicin-encapsulated PEG-b-PLGA copolymeric nanoparticles for targeted photochemotherapy of breast cancer cells

Yu-Hsiang Lee, Da-Sheng Chang

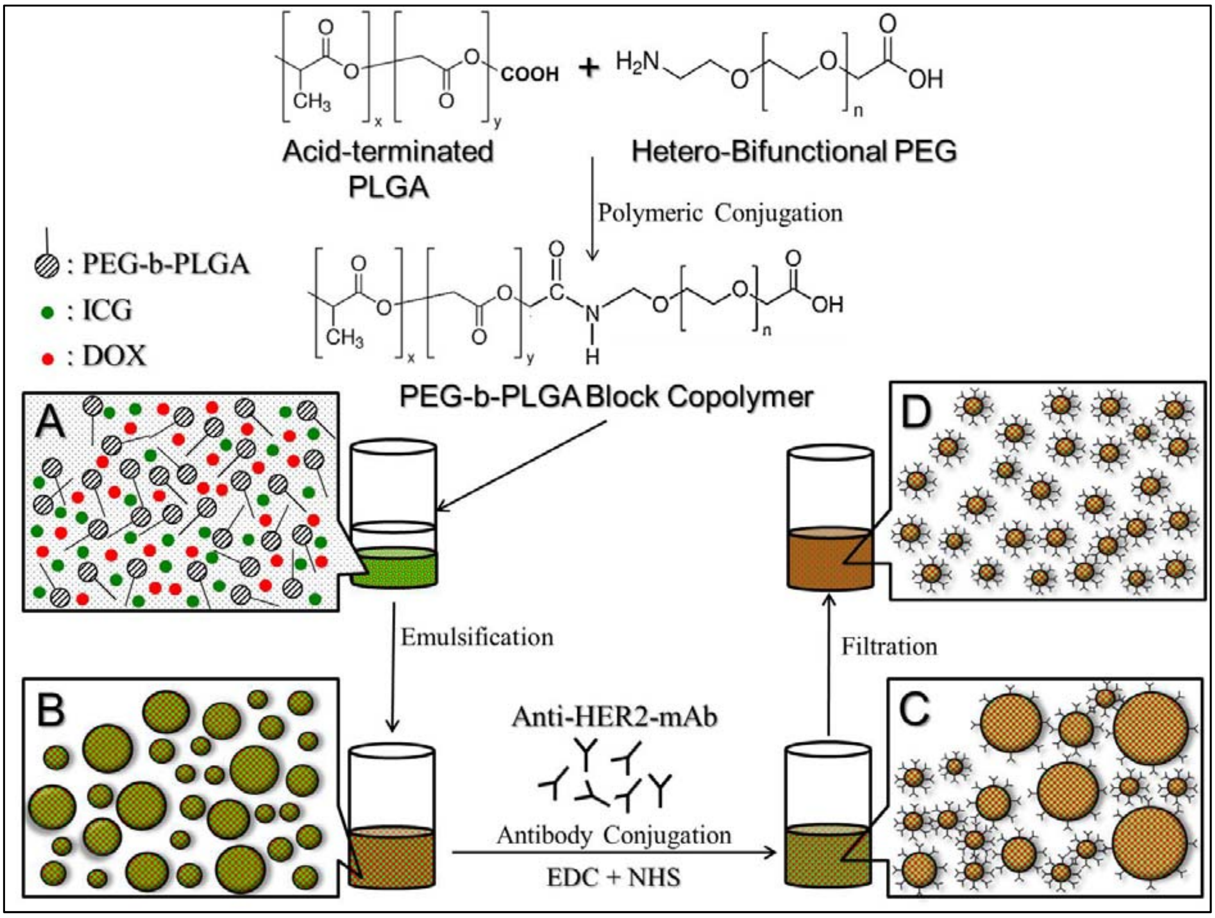

Supplementary Figure S1. **Schematic diagram of the fabrication procedures of the HIDPPNPs.** The PEG-b-PLGA copolymer was first synthesized through carbodiimide chemistry. The PEG-b-PLGA nanoparticles encapsulated with ICG and DOX were then formed using a modified emulsification approach (A → B) followed by conjugation with anti-HER2-mAbs on the nanoparticle surface (C). To remove excess/unreacted chemicals and simultaneously reduce the size dispersity of the products, the produced HIDPPNPs with a broad size distribution were then filtered using a 0.45-μm filter and exhibited improved size uniformity afterward (D).
